# Antimicrobial resistance and virulence gene profiles of *Enterococcus faecalis* and *Enterococcus faecium* isolated from subclinical bovine mastitis milk and cow dung

**DOI:** 10.1038/s41598-025-32569-8

**Published:** 2025-12-18

**Authors:** Ntelekwane George Khasapane, Kgaugelo Lekota, Mofokeng Lehlohonolo, Jane Nkhebenyane, Oriel Thekisoe, Tsepo Ramatla

**Affiliations:** 1 Department of Life Sciences, Centre for Applied Food Safety and Biotechnology, Central University of Technology, 1 Park Road, Bloemfontein, 9300 South Africa; 2 Unit for Environmental Sciences and Management, North-West University, Potchefstroom, 2531 South Africa; 3 Department of Zoology and Entomology, University of the Free State, Phuthaditjhaba, 9866 South Africa; 4 Center for Global Change, University of the Free State, Phuthaditjhaba, 9866 South Africa

**Keywords:** Subclinical mastitis, Enterococci, Antimicrobial resistance, Pathogenesis, Microbiology, Molecular biology

## Abstract

Subclinical mastitis poses a hidden threat to dairy productivity and animal health, often harbouring antimicrobial-resistant pathogens. It is becoming increasingly recognized that *Enterococcus* species cause mastitis in dairy cows. Accurately characterizing the regional epidemiology of enterococcal mastitis, determining its correlations with management variables, and comprehending its effects on udder health all depend on accurate species information. This study investigated the occurrence, antibiotic resistance and virulence factors of *Enterococcus faecalis* and *Enterococcus faecium* in cow dung and milk samples from cows with subclinical mastitis. Subclinical mastitis was identified in 39.0% (68/174) of cows and 27.8% (194/696) of quarters, based on results from the California Mastitis Test (CMT) and somatic cell counts (SCC), respectively. Matrix-Assisted Laser Desorption/Ionization Time-of-Flight Mass Spectrometry (MALDI-TOF-MS) and Polymerase Chain Reaction (PCR) targeting the *ddl* gene confirmed the predominance of *E. faecalis* (93%) and *E. faecium* (6.4%) in milk samples, while cow dung samples yielded only *E. faecalis* (100%). Notably, among the *E. faecalis* isolates from milk samples, 17.2% exhibited vancomycin resistance, whereas streptomycin resistance was found in a smaller proportion of isolates (6.8%). All (100%) *E. faecium* isolates from the same milk samples showed resistance to vancomycin. The findings also revealed that 11 (32.3%) of *E. faecium* isolates from cow dung were resistant to vancomycin. Multidrug resistance (MDR) was observed in 20.6% of milk and 6.8% of cow dung isolates. The *vanA* gene was the most prevalent antibiotic resistance gene (ARG), detected in 96% of *E. faecalis* isolates. Virulence profiling of *Enterococcus* spp. isolates showed varying gene prevalence in milk (*asa1*: 33.3%, *ace*: 12.7%, *esp*: 10%) and cow dung samples (*gelE*: 53.2%, *hyl*: 38.2%). This study has indicated a significant occurrence of antimicrobial-resistant *E. faecalis* and *E. faecium* strains obtained from subclinical cattle mastitis. These findings emphasize the role of *Enterococcus* spp., especially vancomycin-resistant strains, as emerging threats in bovine subclinical mastitis, with possible implications for zoonotic transmission and antimicrobial stewardship in dairy systems.

## Introduction

Bovine mastitis is a major infectious disease of dairy cattle, characterized by physicochemical alterations in milk that compromise its composition and quality. It is considered an endemic disease with significant economic consequences for the global dairy industry^1^. Although antibiotics remain the primary therapeutic option, treatment efficacy is often undermined by the emergence of antimicrobial resistance (AMR), resulting in reduced cure rates and the persistence of opportunistic bacterial infections, including those caused by the *Enterococcus* species^2,3^. Within this genus, *E. faecalis* and *E. faecium* are the most clinically relevant, together accounting for more than 80% of enterococcal infections worldwide. They are recognized as important opportunistic pathogens and are ranked as the third and fourth most common nosocomial agents globally^4,5^. *Enterococcus* species are ubiquitous in nature, capable of contaminating the food chain, colonizing the intestinal tract of both humans and animals, and serving as indicators of faecal contamination in water^6,7^. The predominant species identified in both human and animal microbiota include *E. faecalis*,* E. faecium*,* E. hirae*, and *E. durans*^8^. In dairy cattle, *Enterococcus* can colonize the teat skin and contribute to the pathogenesis of mastitis^9,10^.

Therapeutic management of enterococcal infections is complicated by the high level of resistance to commonly used antibiotics, including bacteremia, endocarditis, urinary tract infections, and pelvic infections^11^. Antibiotic resistance is a natural aspect of bacterial evolution; however, using these compounds in veterinary medicine has accelerated this process by promoting the growth of microorganisms capable of surviving adverse conditions^12^. The bacterial species comprising the ESKAPE group, *Enterococcus* species, *Staphylococcus aureus*,* Klebsiella pneumoniae*,* Acinetobacter baumannii*,* Pseudomonas aeruginosa*, and *Enterobacteriaceae*, are involved explicitly in resistance phenomena^13^. Furthermore, enterococcal strains are becoming resistant to last-resort antibiotics, including daptomycin and linezolid^14,15^.

The pathophysiology of *E. faecalis* is influenced by virulence factors and antibiotic resistance. According to Chajęcka-Wierzchowska et al.^16^, numerous studies have identified virulence factors in *E. faecalis* that may contribute to the severity of infections in humans and animals. Research has documented the existence of virulence factors (*agg* and *ace*) that promote *E. faecalis* adhesion and colonization as well as cytolysis and host dissemination (*cyl*,* gelE*, and *sprE*)^17^. Additionally, *E. faecalis* possesses biofilm-forming machinery (*pili*,* gelE*, and *fsr* quorum-sensing systems) that provides additional antimicrobial resistance and enables the bacterium to cling to biotic and abiotic surfaces^17^. Milk-borne pathogen surveillance in South Africa suffers from limited geographic coverage, reliance on cross-sectional and composite sampling, a lack of longitudinal data, and underrepresentation of smallholder dairy systems. As a result, regions like the Free State’s Thabo Mofutsanyana district remain under-studied. The current study aimed to detect and identify *Enterococcus* spp. from subclinical mastitis cow milk and their associated cow dung samples, including virulence and antibiotic resistance profiles.

## Materials and methods

### Ethical consideration

All procedures and methods were carried out following relevant guidelines and regulations and were reported in accordance with ARRIVE guidelines (https://arriveguidelines.org). No anaesthesia or euthanasia methods were employed or involved in the present study. The study was further authorized by the University of the Free State’s Animal Research Ethics Committee (UFS-AED2024/0015) and the Department of Agriculture, Land Reform and Rural Development by section 20 of the Animal Diseases Act, 1984 (Ref Number: 12/11/1/4/6 (6335 ON)) and all experimental protocols were approved by the two named institutional and/or licensing committee .

### Study design, sampling strategy and initial screening of SCM

**Fig. 1:**
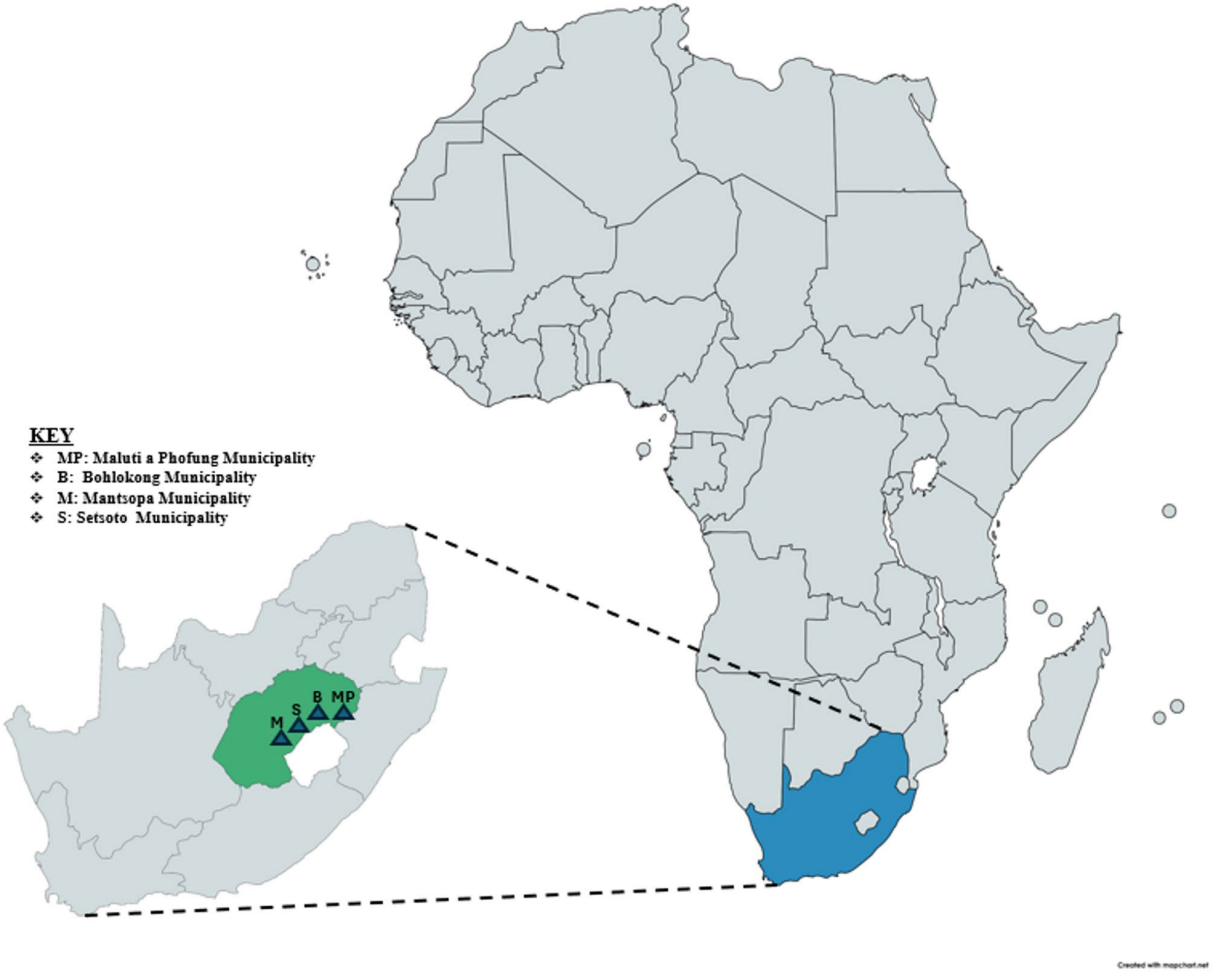
Geographical locations of sample collection sites within the Free State Province, South Africa [(https://www.mapchart.net/world.html (accessed on 02 May 2025)].

In this cross-sectional study, the authors collected 174 milk samples from dairy cows across six farms in the Thabo Mofutsanyana District Municipality, Free State Province (Fig. 1). A total of 174 composite milk samples were collected using 50 mL sterile containers from four local municipalities: Mantsopa (40), Bohlokong (40), Maluti-a-Phofung (40), and Setsoto (54). Following the National Mastitis Council’s (NMC) guidelines, aseptic milk samples were obtained from cows that appeared to be healthy, from each quarter.

A cow was screened for subclinical mastitis on the farm using the California Mastitis Test (CMT). Before sampling, each cow’s udder was cleaned with distilled water and dried with a disposable paper towel to prevent cross-contamination. After cleaning the udder and disinfecting the teats with 70% ethanol, 10 ml of milk was drawn from each cow. Next, 2 mm of milk samples from each lactating quarter that were free of clinical mastitis were milked and put into different cups on the CMT paddle. By tilting the CMT paddle and decanting the extra milk, the milk samples were equalized. Each cup of the CMT paddle containing the milk received an equal amount of CMT reagent, which was well mixed for 15 s. Thereafter, positive cow samples were then checked for SCM using the somatic cell count (SCC) assay (Mérieux NutriSciences, South Africa) and using Karzis et al.^18^ recommended scoring and interpreting of the level of inflammation based on the CMT and SCC results were scored as follows: 0 (negative (healthy quarter), somatic cell count (SCC) ≤ 100,000 cells/mL milk), 1+ (weak positive, SCC > 100,000 < 500,000 cells/mL milk), 2+ (distinct positive, SCC > 500,000 < 1000,000 cells/mL milk), and 3+ (strong positive, SCC ≥ 1000,000 cells/mL milk). Furthermore, faecal samples were collected directly from the rectum of each animal deemed to have subclinical mastitis and placed in a 50 mL sterile container. All samples were processed within 12 h of collection to ensure optimal preservation and integrity of the specimens^19^. Before the analysis, the samples were thawed in a refrigerator.

### Isolation and identification of *Enterococcus* species

After plating 100 µL of milk onto Slanetz and Bartley Agar (SBA) (Oxoid, UK), the samples were incubated at 37 °C for 24 to 48 h. The sample culture was considered negative if no bacteria could be isolated after the direct plating procedure. A total of 68 cow dung samples from subclinically ill cows (10 g) were homogenized in 90 mL of a 2% sterile Buffered Peptone water (Oxoid, UK) for 2 min in a Stomacher BagMixer (Interscience, St. Nom, France). Samples were manually subjected to serial dilutions up to 10^–8^ dilution factor and then plated on Slanetz and Bartley agar (Oxoid, UK), followed by incubation at 37 °C for 24–48 h. The selection of enterococcal strains was aided by developing dark reddish colonies on the surface of the utilized media following cultivation. Following double purification, pure cultures from distinct colonies were identified as potential *Enterococcus* species.

The isolates were then identified using matrix-assisted laser desorption ionization-time of flight (MALDI-TOF) mass spectrometry with MALDI-Biotyper 3.0 software (Bruker Daltonics, Bremen, Germany), as previously reported by Cameron et al.^20^. Two identification attempts were performed for each isolate. Isolates were considered unidentified if they could not be identified after two rounds of MALDI-TOF MS analysis. A cut-off score of ≥ 1.7 was used as the threshold for quality control. For further research, all pure *Enterococcus* spp. isolates were stored at -80 °C in Brain Heart Infusion (BHI) broth with 15% glycerol.

### Genomic DNA extraction of *Enterococcus* species

A colony grown overnight at 37 °C on nutrient agar plates had its DNA extracted. Each colony was vortexed for two minutes after being inoculated into 200 µL of sterile distilled water. Thermo Fisher Scientific, Germany, centrifuged the cells for ten minutes at 13,000 rpm. The cells were lysed for 15 min at 100 °C in a heat block following the pipetting of 500 µL of distilled water into the Eppendorf tubes and vortexing^21^. The cell debris was separated using a Mini Spin centrifuge (Thermo Fisher Scientific, USA), which centrifuged the same sample for five minutes at 10,000 rpm. The supernatant was used immediately as a PCR template after extraction.

### Molecular identification of the isolates

Species identification of Enterococci was further confirmed using singleplex-PCR targeting the species-specific *ddlE* gene. The primers employed were specific to *E. faecium* (550 bp amplicon; F: TAGAGACATTGAATATGCC, R: TCGAATGTGCTACAATC) and *E. faecalis* (941 bp amplicon; F: ATCAAGTACAGTTAGTCT, R: ACGATTCAAAGCTAACTG), as previously described by Jannati et al.^22^. The *Enterococcus* spp. DNA template (1 µL), 10 µL of Taq 2X Master Mix from New England Biolabs, which is distributed by Inqaba Biotech (South Africa), 1 µM per primer (1 µL), and 7 µL of RNase water made up a total of 20 µL PCR reaction. The PCR amplifications were conducted following the established method using a thermal cycler (Eppendorf^®^ Mastercycler Personal). The cycling parameters for PCR consisted of an initial denaturation step at 94 °C for 2 min, followed by 30 cycles of denaturation at 94 °C for 1 min, annealing at 54 °C for 1 min, extension at 72 °C for 1 min, and a final extension step at 72 °C for 10 min. Both *E. faecalis* ATCC 29212 and *E. faecium* ATCC 700221 strains were utilized as positive controls. The PCR amplicons (5 µL) were analysed in a 1.2% agarose gel electrophoresis using ethidium bromide staining (0.5 µg/mL) and at 100 volts for 30 min. PCR analysis was used to identify *Enterococcus* spp., with *E. faecalis* species yielding a 941 bp product and *E. faecium* species yielding a 550 bp product. Moreover, due to budgetary constraints, the PCR products were not sent for sequencing to obtain their accession numbers.

### Molecular detection of *Enterococcus* species using virulence genes

Six virulence genes were screened among the *Enterococcus* isolates (Table 1). These included genes associated with aggregation substances (*asa1*), enterococcal surface protein (*esp*), gelatinase (*gelE*), glycosyl hydrolase (*hly*), cytolysin (*cylA*), and genes encoding collagen-binding protein (*ace*).

**Table 1:** Primer sequences of antimicrobial-resistant and virulent genes screened among *Enterococcus* species.

	Genes	Primer sequence (5´-3´)	Annealing temp (°C)	Amplicon size (bp)	References
AMR genes	*tetK*	TCGATAGGAACAGCAGTA	55 °C	844	Bag et al.^23^
		CAGCAGATCCTACTCCTT			
	*tetL*	TCGTTAGCGTGCTGTCATTC	55 °C	267	
		GTATCCCACCAATGTAGCCG			
	*tetM*	GTGGACAAAGGTACAACGAG	55 °C	406	
		CGGTAAAGTTCGTCACACAC			
	*tetS*	CATAGACAAGCCGTTGACC	55 °C	667	
		ATGTTTTTGGAACGCCAGAG			
	*int*	GCGTGATTGTATCTCACT	50 °C	1028	Morandi et al.^24^
		GACGCTCCTGTTGCTTCT			
	*ermB*	CATTTAACGACGAAACTGGC	55 °C	738	Jensen et al.^25^
		GGAACATCTGTGGTATGGCG			
	*vatD*	GCTCAATAGGACCAGGTGTA	58 °C	361–632	Jensen et al.^22^
		TCCAGCTAACATGTATGGCG			
	*vatE*	ACTATACCTGACGCAAATGC	58 °C	361–632	
		GGTTCAAATCTTGGTCCG			
	*vanA*	TCTGCAATAGAGATAGCCGC	55 °C	377	Jensen et al.^22^
		GGAGTAGCTATCCCAGCATT			
	*vanB*	GCTCCGCAGCCTGCATGGACA	58 °C	529	
		ACGATGCCGCCATCCTCCTGC			
Virulence genes	*asa1*	GCACGCTATTACGAACTATGA	56 °C	375	Morandi et al.^24^
		TAAGAAAGAACATCACCACGA			
	*esp*	AGATTTCATCTTTGATTCTTGG	56 °C	510	
		AATTGATTCTTTAGCATCTGG			
	*gelE*	TATGACAATGCTTTTTGGGAT	56 °C	213	
		AGATGCACCCGAAATAATATA			
	*hyl*	ACAGAAGAGCTGCAGGAAATG	56 °C	276	
		GACTGACGTCCAAGTTTCCAA			
	*cylA*	ACTCGGGGATTGATAGGC	55 °C	688	
		GCTGCTAAAGCTGCGCTT			
	*ace*	GAATTGAGCAAAAGTTCAATCG	55 °C	1008	
		GTCTGTCTTTTCACTTGTTTC			

### Antimicrobial susceptibility testing

Antimicrobial susceptibility testing was performed using the disc diffusion method on Mueller-Hinton agar (Merck, Germany). The discs were put on a plate and incubated aerobically for 18 to 24 h at 37 °C following spreading the overnight inoculum containing the isolates. The antimicrobial susceptibility test results were interpreted according to the guidelines set by the Clinical and Laboratory Standards Institute (CLSI)^26^. Eight antibiotics, which were acquired from Thermo Fischer ScientificTM (Thermo Fisher, South Africa), were employed in this investigation: Ampicillin (10 µg), Ceftazidime (µg), Erythromycin (15 µg), Gentamicin (10 µg), Tetracycline (30 µg), Vancomycin (µg), Chloramphénicol (µg), and Streptomycine (10 µg). Antimicrobial resistance was determined using *Enterococcu*s *faecalis* ATCC 29212 and *E. faecium* ATCC 700221 as the positive control. According to CLSI criteria, plates were interpreted as positive when growth of *Enterococcus* was observed within the inhibition zone adjacent to the antibiotic disk.

### Molecular detection of antibiotic-resistant genes

The presence of genes associated with tetracycline, erythromycin, quinupristin/dalfopristin, and vancomycin was examined for each strain using specific PCR assays. Ten antibiotic-resistant genes were screened in this study, including tetracycline-resistant genes encoding ribosomal protection proteins (*tetM* and *tetS*) and efflux proteins (*tetK* and *tetL*), the transposon integrase gene (*int*) of the Tn916/Tn1545 family, erythromycin resistance gene (*ermB*), and vancomycin resistance genes (*vanA* and *vanB*) (Table 1).

### Evaluation of MAR index, MDR, and XDR patterns in bacterial isolates

The formula described by Rajendiran et al.^27^, was used to calculate the multiple antibiotic resistance (MAR) index. MAR = (total number of antibiotics tested) / (number of antibiotics displaying resistance by an isolate). Values near 0 indicated more sensitivity, while values near 1 indicated significant resistance. A MAR score of 0.20 or higher suggested a significant resistance level or a high-risk reservoir of bacterial contamination. A high-risk reservoir of bacterial contamination in terms of MAR refers to an environment, host, or material that harbours bacteria and supports the persistence and dissemination of multidrug-resistant strains. Such reservoirs act as amplifiers and transmission hubs of resistant organisms, posing significant threats to public health and food safety. Additionally, MDR is characterized by nonsusceptibility to at least one agent in three or more categories of antimicrobials.

### Statistical analysis

Descriptive statistics, particularly proportions, percentages, means, and two-way ANOVA and Fisher’s exact test, were calculated using Excel software (2013) with *P* ≤ 0.05 considered significant.

## Results

### California mastitis test (CMT) and somatic cell counts (SCC)

Out of the 174 cows that were sampled, only 68 (39.0%) were positive for subclinical mastitis at a cow level based on CMT. Moreover, only 194/696 (27.8%) quarter-level samples were positive for subclinical mastitis based on the SCC assay.

### Identification and detection of *Enterococcus* spp. Isolates

**Fig. 2:**
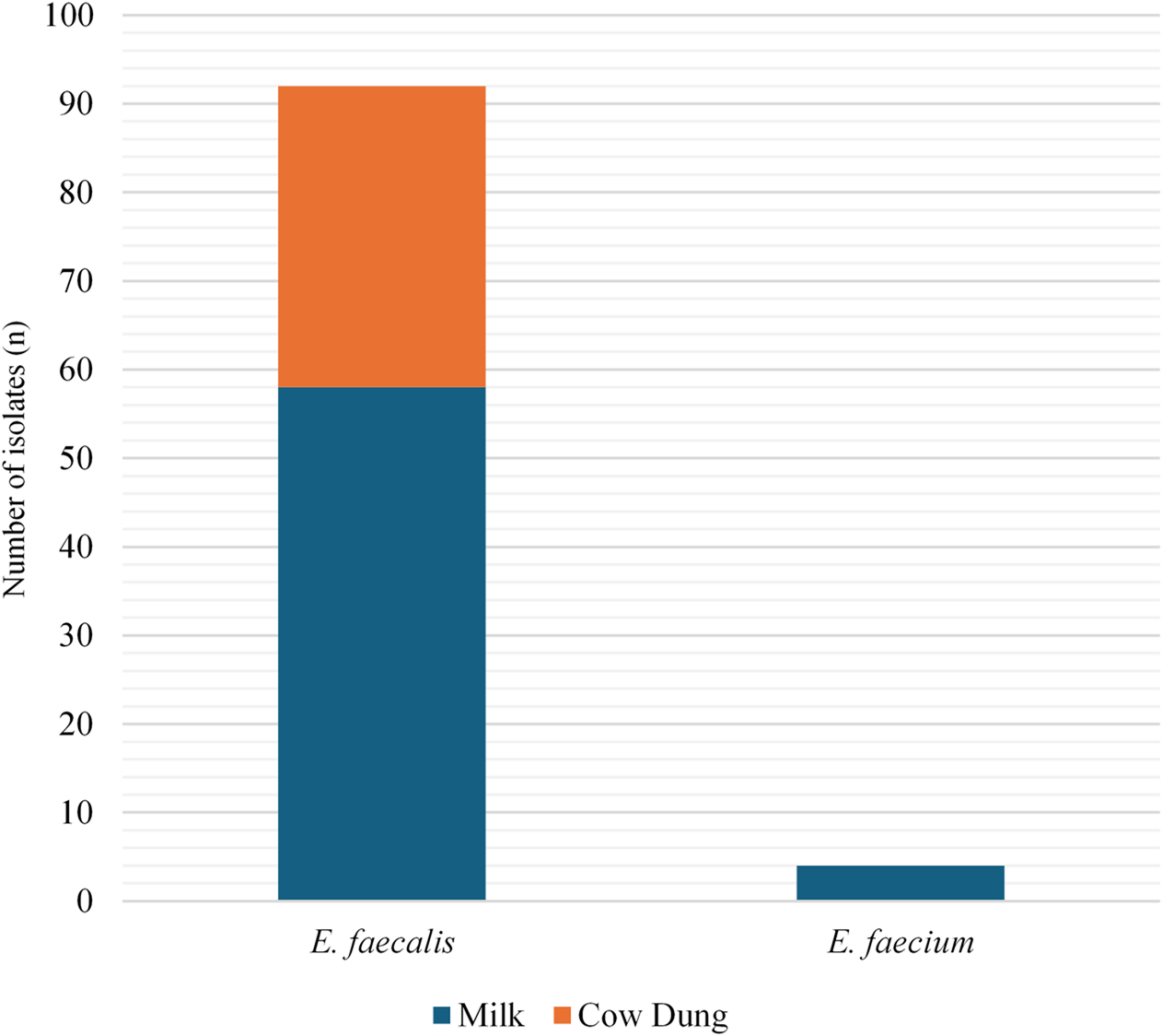
The number of *Enterococcus* species isolates cultured from milk and cow dung samples from dairy cows with subclinical mastitis.

A total of 62 presumptive *Enterococcus* strains were isolated from 68 milk samples that were considered to have subclinical mastitis from 174 dairy cows across six farms. MALDI-TOF-MS analysis and *dd1* species-specific gene PCR assays confirmed the identification of all 58 (93%) *E. faecalis* and 4 (6.4%) *E. faecium* isolates from milk samples. The 0.6% remaining were all negatives and did not amplify by species-specific genes. In contrast, the study isolated 34 presumptive *Enterococcus* species from cow dung, with all (100%) identified as *E. faecalis.* All confirmed isolates in this study are shown in Fig. 2. Furthermore, using Fisher’s exact test, our study found that there’s no statistically significant association between sample type (Milk vs. Cow Dung) and the presence of *E. faecalis* vs. *E. faecium* (*p*-value: 0.294).

### Phenotypic antimicrobial resistance

The results of the antimicrobial resistance analysis using the disc diffusion method for *E. faecalis* and *E. faecium* isolates from bovine milk and cow dung are presented in Table 2. Among all 58 *E. faecalis* isolates identified from milk samples, 10 (17.2%) showed resistance to vancomycin, followed by tetracycline, ceftazidime, erythromycin, and streptomycin at 7 (12.0%), 6 (10.3%), 6 (10.3%), and 4 (6.8%), respectively. All *E. faecium* (100%) isolates from milk samples were resistant to vancomycin, with resistance also observed to streptomycin, erythromycin, and ceftazidime at 3 (75%), 2 (50%), and 2 (50%), respectively. Furthermore, the results indicated that 11 (32.3%) of the *E. faecalis* isolates from cow dung were resistant to vancomycin, followed by resistance to ceftazidime, tetracycline, and ampicillin, with 7 (20.5%), 5 (14.7%), and 5 (14.7%), respectively. The statistical analysis revealed that for both *E. faecalis* and *E. faecium* from milk samples *p*-value was 0.001, while isolates from cow dung samples had a p-value of 0.01, indicating statistically significant results between the number of isolates from each sample and the resistance proportion of antibiotics used.

**Table 2:** Antimicrobial resistance profiles of *Enterococcus faecalis* and *E. faecium* isolated from milk and cow dung samples.

Antibiotics	Samples			
	Milk		Cow dung	
	E. faecalis	E. faecium	E. faecalis	E. faecium
Vancomycin	10 (17.2%)	4 (100%)	11 (32.3%)	-
Gentamycin	2 (3.4%)	-	2 (5.8%)	-
Tetracycline	7 (12.0%)	-	5 (14.7%)	-
Ceftazidime	6 (10.3%)	2 (50%)	7 (20.5%)	-
Streptomycin	4 (6.8%)	3 (75%)	1 (2.9%)	-
Chloramphenicol	3 (5.1%)	-	4 (11.7%)	-
Ampicillin	2 (3.4%)	-	5 (14.7%)	-
Erythromycin	6 (9.6%)	2 (50%)	0 (0%)	-
*p*-value	0.001		0.01	

### The multidrug-resistant profile of the *E. faecalis* and *E. faecium* isolates

The results showed that 16 (25.6%) of *E. faecalis* and *E. faecium* isolates were classified as multidrug-resistant. Six (10.3%) *E. faecalis* and 6 (10.3%) *E. faecium* isolates from milk were categorized as MDR, respectively. Only 4 (6.8%) *E. faecalis* isolates from cow dung samples were MDR. The multidrug-resistance patterns of the isolates are shown below (Table 3).

**Table 3:** Multidrug-resistant profiles of *E. faecalis* and *E. faecium* isolates from milk and cow Dung samples.

AMR phenotypes	Milk		Cow dung
	E. faecalis	E. faecium	E. faecalis
CAZ, S, AM	1	-	-
C, AM, E	-	-	-
TE, S, C	-	1	1
VA, GEN, TE	-	-	1
VA, TE, C	-	1	1
VA, TE, CAZ	1	-	-
VA, CAZ, S	-	-	-
VA, CAZ, AM	1	1	4
VA. TE, S, E	-	-	-
VA, TE, CAZ, S, E	-	-	-
VA, TE, C, AM	1	-	1
VA, GEN, TE, C	1	2	1
VA, GEN, TE, CAZ, E	-	1	-
VA, GEN, C, AM, E	1	-	-
Total	6	6	4

### The MAR index of the resistant isolates

Table 4 of the MAR results shows that the 19 *Enterococcus* isolates cultured from milk and cow dung exhibit significant antibiotic resistance.

**Table 4:** MAR index of the *Enterococcus* isolates from milk and cow dung.

Isolate no	No. of antibiotics to which isolate was resistant (a)	MAR index = a/b
M1	4	0.5
M2	5	0.6
M3	3	0.3
M4	5	0.6
M5	3	0.3
M6	3	0.3
M7	3	0.3
M8	3	0.3
M9	3	0.3
M10	5	0.6
CD1	3	0.3
CD2	4	0.5
CD3	3	0.3
CD4	3	0.3
CD5	4	0.5
CD6	3	0.3
CD7	3	0.3
CD8	3	0.3
CD9	3	0.3

### Molecular detection of antimicrobial-resistant genes

**Fig. 3:**
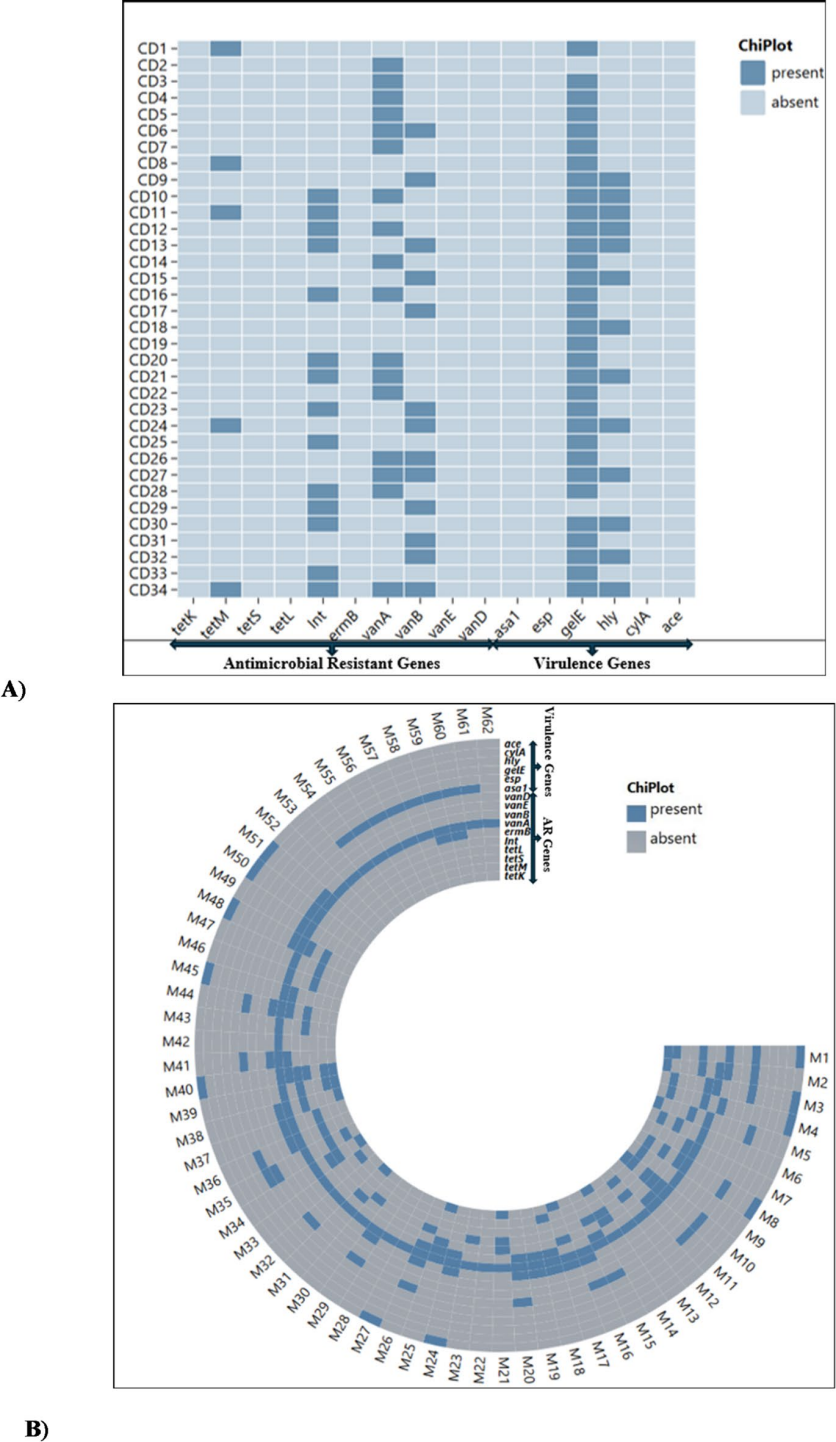
Heatmap illustrating the clustering of antimicrobial-resistant genes and virulence genes detected from cow dung (**A**) and milk samples (**B**) derived *Enterococcus* spp. isolates.

The genomic detection of antimicrobial resistant genes (ARGs) revealed that the majority of all isolates harboured the *vanA* gene, with 60 (96%), followed by *tetM*,* ermB*,* vanB*, and *tetL*, each present in 20 (62.5%) isolates. Additionally, 13 (20.9%) isolates from milk samples carried the *tetk* gene. Of these isolates, 56 (93.3%) harbouring the *vanA* gene were *E. faecalis*, while 4 (6.6%) were *E. faecium*. Furthermore, 20 (62.5%) of *the tetM* isola*tes and* 17 (85%) of the *tetk* isolates were *E. faecalis*, with the remaining 3 (15%) being *E. faecium*. The *vanB* gene was found exclusively in *E. faecalis*. The genomic results also showed that, from cow dung samples, *vanA* was the most frequently detected gene, present in 17 (50%) isolates. This was followed by *vanB* and *tetM*, with 13 (38.2%) and 5 (8.3%) isolates, respectively. None of the isolates from cow dung carried the *ermB*,* tetL*,* tetS*,* vanD*, or *vanE* genes. Interestingly, the transposon integrase gene (*int*) was detected in 29 (87%) *E. faecalis* strains and 4 (12.1%) *E. faecium* strains from milk samples. In comparison, 15 (46.4%) of the *E. faecalis* isolates from cow dung also showed the presence of the transposon integrase gene (*int*) (Fig. 3A and B).

### Molecular detection of virulence genes

The results of the virulence profile further showed that 20 (33.3%) of the isolates from milk samples harboured the *asa1* gene, followed by the *ace* and *esp* genes with 11 (12.7%) and 6 (10%) respectively. Moreover, none of the isolates exhibited detection of the *hyl* and *cylA* genes. Of the 20 isolates with the *asa1* gene detected, 17 (85%) were *E. faecalis*, while 4 (20%) were *E. faecium*. Furthermore, 10 (90.9%) isolates with the ace gene were *E. faecalis*, and 1 (10%) was *E. faecium*. The *esp* gene was detected in *both E. faecalis* and *E. faecium*, with 3 (50%) each. In addition to milk samples, only two virulence genes, *gelE* and *hyl*, were detected in isolates from cow dung samples, with 33 (53.2%) and 13 (38.2%) isolates, respectively. A total of 30 (90.9%) isolates carrying the *gelE* gene were *E. faecalis*, while the remaining 3 (9.0%) were *E. faecium*. Moreover, all 13 (38.2%) isolates carrying the *hyl* gene were *E. faecali*s (Fig. 3A and B).

### Coexistence of phenotypic and genotypic antibiotic resistance among *E. faecium* and *E. faecalis*

This study further showed that 8 out of 59 (13.5%) strains of *E. Faecalis* and 4 (100%) strains of *E. Faecium* from milk samples exhibited a coexistence of phenotypic and genotypic antimicrobial resistance (AMR) and virulence factors (Table 5). In contrast, all four strains (11.7%) of *E. faecalis* isolated from cow dung demonstrated the same coexistence of antimicrobial resistance and virulence factors (Table 6).

**Table 5:** Co-occurrences of resistance phenotypes, genotype, and the prevalence of *Enterococcus* strains from milk samples.

E. faecalis				E. faecium			
Isolates ID:	Phenotypic	AMR genes	Virulence genes	Isolates ID:	Phenotypic	AMR genes	Virulence genes
MS2	VA, TE, E, S, CAZ,	*vanA*, *tetM*, *ermB*, *vanB*, * tetL*,* tetK*,* int*	*Asa1*, *ace*, *esp*	MS35	VA, S, E, CAZ	*Int vanA*,* tetM*	*Esp*,* Asa1*
MS3	VA, TE, E, S, CAZ	*vanA*, *tetM*,* ermB*,*vanB*, * tetL*,* tetK*,* int*	*Asa1*, *ace*	MS36	VA, S, E, CAZ	*Int*, *vanA*,* tetM*	*Asa1*
MS4	VA, TE, E, S, CAZ		*Asa1*,* ace*,* esp*	MS41	VA, S	*Int*,* vanA*,* tetm*	*Asa1*
MS10	VA, TE, E, CAZ	*vanA*,* tetM*,* ermB*,* vanB*, * tetL*,* tetK*,* int*	*Asa1*,* ace*,* esp*	MS44	CA	*Int*,* vanA*	*Asa1*
MS25	VA, TE, E, CAZ,	*vanA*,* tetM*,* ermB*,* vanB*, * tetL*,* tetK*,* int*	*Asa1*,* ace*,* esp*	-	-	*-*	*-*
MS26	VA, TE	*vanA*,* tetM*,* ermB*,* vanB*, * tetL*,* tetK*,* int*	*Asa1*,* ace*,	-	-	*-*	*-*
MS27	VA	*vanA*,* tetM*,* ermB*,* intvanB*, *tetL*,* tetK*,* int*	*Asa1*,* ace*	-	-	*-*	*-*
MS29	VA	*vanA*,* tetM*,* ermB*,* vanB*, * tetL*,* tetK*,* int*	*Asa1*,* ace*	-	-	*-*	-

*VA, TE, E, S, CAZ, GEN, C, AMP: Vancomycin, Tetracycline, Erythromycin, Streptomycin, Ceftazidime.

**Table 6:** Co-occurrences of resistance phenotypes, genotype, and the prevalence of *Enterococcus* strains from cow Dung samples.

E. faecalis			
Isolates ID:	Phenotypic	AMR genes	Virulence genes
CD10	VA, CAZ, TE	*vanA*,* VanB*,* tetM*,* int*	*gelE*,* hyl*
CD12	VA, CAZ, TE, AMP	*VanA*,* vanB*,* tetM*,* int*	*gelE*,* hyl*
CD21	VA, CAZ, TE, AMP	*vanA*,* vanB*,* tetM*,* int*	*gelE*,* hyl*
CD34	VA, CAZ, TE, AMP	*vanA*,* vanB*,* tetM*,* int*	*gelE*,* hyl*,* esp*,* ace*

*VA, TE, E, S, CAZ, GEN, C, AMP: Vancomycin, Tetracycline, Erythromycin, Streptomycin, Ceftazidime.

## Discussions

A comparison with earlier studies conducted in similar management systems in Sri Lanka, which reported SCM prevalence rates of 43.0% at the cow level and 49.0% at the quarter level^28,29^, reveals that the current study found slightly lower prevalence rates of 39.0% at the cow level and 27.8% at the quarter level, respectively. Nonetheless, similar prevalence levels were discovered in crossbred dairy cow herds in Bangladesh that were managed using a semi-intensive system^30^ and in equally hot tropical parts of Mexico^31^.

In contrast to the current study, other research has found higher prevalence rates of SCM in smallholder dairy farms with both grazing and zero grazing in Kampala, Uganda^32^, as well as under intensive management systems in southern Brazil^33^ and Poland^34^. Because of risk factors such low grazing, poor udder hygiene, increased parity, and late lactation, Abrahmsén et al.^32^, found an abnormally high prevalence of SCM in Uganda (86.2%). Although the incidence of SCM in Zimbabwe’s smallholder dairy farms was relatively low (16.3%)^35^, defined SCM as having SCC of at least 300 × 10^3^ cells/mL.

Enterococci are one of the environmental pathogens responsible for bovine mastitis, and antimicrobials are widely used to treat these infections^9^. Unfortunately, *Enterococcus* has inherent resistance to many broad-spectrum antibiotics, which poses a significant threat to public health. Understanding the enterococci’s prevalence, antimicrobial resistance, and virulence factors is crucial for targeted therapy and control of bovine mastitis. These findings align with research showing that *E. faecalis* is more frequently isolated from animal sources and dairy environments than *E. faecium*^36,37^. According to Montealegre et al.^38^, the prevalence of *E. faecalis* in cow manure may be due to its adaptation to the cattle’s digestive tract, where it persists as a commensal microbiota. Since *E. faecalis* and *E. faecium* are opportunistic pathogens capable of surviving in various conditions, their presence in milk samples may indicate contamination during milking or subsequent handling^7^.

*Enterococcus* isolates from milk and cow dung samples exhibited distinctive patterns in their virulence gene profiles, which could indicate their ability to cause disease and adapt to their environment. The *asa1* gene was most common among milk isolates, present in 33.3% of samples, mainly *E. faecalis* (85%). According to Gilmore et al.^39^, the *asa1* gene encodes an aggregating substance that boosts colonisation and infection capabilities by encouraging adhesion and biofilm development. This prevalence emphasizes the significance of the *asa1* gene in the pathogenesis of infections in dairy cattle. Saeed et al.^40^, noted that similar levels of *asa1* were found in *E. faecalis* from the European gut microbiome of healthy individuals. The *ace* gene, which encodes a collagen-binding adhesin necessary for tissue attachment and persistence, was found in 12.7% of milk isolates (mostly *E. faecalis*) in Germany^41^. The *esp* gene, distributed equally between *E. faecalis* and *E. faecium* and present in 10% of milk isolates, is well known for its role in immune evasion and biofilm formation in Serbia^42^. While a study by Rahman et al.^43^, in Bangladesh has reported the presence of *cylA* and *hyl* genes in bovine-derived enterococci, their absence in milk isolates may suggest that the distribution of virulence genes varies depending on regional factors or management practices. Only the virulence genes *gelE* (53.2%) and *hyl* (38.2%) were found in cow dung samples. The *hyl* encodes hyaluronidase, facilitating bacterial spread through host tissues, while *gelE* encodes gelatinase, aiding tissue breakdown and immune evasion^44^. The nearly exclusive presence of both genes in *E. faecalis* suggests that this species is more virulent and better adapted to its environment than *E. faecium*^45^. Manure may serve as an environmental reservoir for virulent enterococci, potentially posing risks to both animal and public health, as evidenced by detecting these genes in dung^7^.

The findings show that *Enterococcus* isolates from dairy sources, especially *E. faecalis* and *E. faecium*, have an antimicrobial resistance pattern. Notably, isolates from milk and cow dung had isolates which were resistant to vancomycin in this study. This is consistent with recent research showing that *E. faecium* is more often multidrug-resistant than *E. faecalis*^46^. Serious worries regarding zoonotic transmission through the food chain and environmental exposure are raised by the high level of resistance to vancomycin, a last-resort antibiotic in human medicine^7^. The extensive use of tetracycline, erythromycin, and streptomycin in cattle management, particularly in developing nations with less stringent regulations, is also reflected in the observed resistance to these antibiotics^47^. Due to their historical usage of tetracycline and macrolides (such as erythromycin) medicinal drugs, *Enterococcus* species isolated from dairy cattle frequently resist them^48^. Antimicrobial-resistant *Enterococcus* in cow dung points to an environmental reservoir of resistance genes that may be made worse by inappropriate manure disposal and ecosystem damage^37^. The discovery of ampicillin and ceftazidime resistance emphasizes the development of resistance mechanisms, which may have occurred due to horizontal gene transfer between bacterial genera or enterococci^49^.

As previously noted, the *vanA* gene is the primary genetic factor responsible for vancomycin resistance in enterococci isolated from animal sources. The high prevalence of *vanA*, particularly among *E. faecalis* (93.3%) compared to *E. faecium* (6.6%), supports these findings^39^. Interestingly, the *vanB* gene was only found in isolates of *E. faecalis*. This aligns with research by Arredondo-Alonso et al.^50^, which showed that *vanB* carriage is less consistent and often linked with *Enterococcus* strains from Dutch hospitals. The widespread use of tetracyclines in veterinary medicine, which exerts selection pressure favouring the retention of such resistance determinants, is consistent with the high detection rates of tetracycline resistance genes *tetM* (62.5%) and *tetK* (20.9%) in milk isolates as shown by a global study of Barel et al.^48^. Nowakiewicz et al.^51^ in Poland observed that the co-occurrence of the *tetM* gene and the int integrase gene from the Tn916/Tn1545 transposon family in many *E. faecalis* strains highlights the role of mobile genetic elements in promoting horizontal gene transfer of tetracycline resistance. This was present in 87% of milk samples and 46.4% of dung samples. It is widely recognized that this integrative conjugative element facilitates the spread of resistance within and between bacterial populations in cattle contexts^49^.

Interestingly, *ermB*, which causes resistance to macrolides, was found in milk isolates at a frequency comparable to *tetM* but not in dung isolates. Agga et al.^52^, observed niche-specific ARG distributions in cow farms, which could reflect variations in antibiotic exposure or selection pressures in different habitats. The absence of several ARGs (*ermB*,* tetL*,* tetS*,* vanD*, and *vanE*) in cow dung isolates highlights differences in the resistome between host-associated and environmental microbiota, possibly due to manure management techniques^37^. These findings support the idea that resistance genes form a dynamic and complex reservoir in dairy farm settings, with implications for spreading AMR to the broader environment and public health^43^.

The MDR to antibiotics such as vancomycin, tetracycline, ceftazidime, erythromycin, and streptomycin was found in 20.6% of *Enterococcus* isolates from milk samples and 6.8% from cow dung. These findings are consistent with recent developments in veterinary microbiology. Studies conducted worldwide have raised concerns regarding treatment effectiveness and zoonotic transmission, showing the rising incidence of MDR enterococci in animals used for food production. For example, the 20.6% MDR rates discovered in milk isolates in this study closely matched the 25% MDR rates found in *Enterococcus* isolates from bovine mastitis in a survey by Saeed et al.^40^. Similar to but marginally higher than the 6.8% MDR found in cow dung samples in the current investigation, Rahman et al.^43^, reported MDR rates ranging between 10 and 15% in enterococci from cattle manure in Bangladesh, which may be due to regional or farm management variations.

The MAR indices are routinely calculated to assess health concerns, particularly when antibiotic use is rare, and the risk of transmission to the community could have significant health implications^53^. In this study, the MAR indices were much higher than expected, indicating that treating infections associated with these strains is highly challenging. Similar to the findings of Adeyemi et al.^54^, most organisms exhibited values above 0.2, suggesting that these isolates are indeed high-risk pathogens originating from environments with frequent antibiotic use.

Additionally, this investigation demonstrated that virulence factors and phenotypic and genotypic antimicrobial resistance (AMR) coexisted in 8 out of 59 (13.5%) strains of *E. Faecalis* and 4 (100%) strains of *E. Faecium* from milk samples. On the other hand, all four of the *E. faecalis* strains (11.7%) that were isolated from cow dung showed the similar coexistence of virulence factors and antibiotic resistance. Furthermore, given that some of these antibiotic-resistant medications work well against isolates that form biofilms, it is intriguing that *asa1*,* gelE*, and *esp* genes have a positive correlation with resistance profiles^55^. This is why we believe that exposure to antibiotic-resistant medications may cause biofilm-forming isolates to develop a particular resistance to these substances. Antibiotic-resistant bacteria benefit from the connection of asa1, *esp*, and *gelE* since it gives them two weapons to cause and survive clinical infection treatment^55^. Additionally, the findings demonstrated the discrepancy between genotypic and phenotypic AMR. Notwithstanding the benefits of molecular approaches, the capacity of molecular panels to forecast susceptibility is entirely reliant on their target list and our comprehension of resistance processes. Because the mechanism of AMR may not be included as a target on molecular panels, the absence of an AMR marker does not always indicate susceptibility for certain antimicrobials^56^. The majority of clinically meaningful resistance in Gram-positive organisms is often caused by a single mechanism of resistance (*van*A/B for VRE), and forecasts of susceptibility profiles have been reported to have accuracy rates ranging from 98 to 100%^57,58^.

Risk factors for subclinical mastitis, including advanced age and higher parity, specific udder and teat conformations, such as enlarged udders, poor teat end health (hyperkeratosis), and high udder dirtiness, were not taken into account in the current study. High humidity, poor herd cleanliness and sanitation, poorly maintained or insufficient milking equipment, particular housing systems, lengthier lactation stages, and inconsistent or insufficient post-milking teat disinfection are some environmental and management factors that enhance risk.

## Conclusion

This study enhances our understanding of the enterococcal communities in raw milk and cow dung. Our findings indicate that cow dung does not serve as a source or potential reservoir for antimicrobial-resistant (AR) enterococci in the milk microbiota, as these microorganisms originate from different habitats than those found in milk. We hypothesize that farm surfaces or milking equipment may be the primary sources of enterococci in milk. It is also essential to consider the significant presence of multidrug-resistant (MDR) isolates and their capacity to spread AR genes through dairy products. Our research confirms a notable prevalence of vancomycin-resistant enterococci (VRE) in the dairy environment of South Africa, which is evident from the substantial presence of VRE in both milk and dung samples. To the best of our knowledge, this study is the first to investigate the relationship between the origin, pathogenicity, and antibiotic resistance of enterococci isolated from the milk of cows with subclinical mastitis and the corresponding bovine dung in the Free State Province, South Africa.

## Data Availability

The data supporting the findings of this study are included in the manuscript. Moreover, due to budgetary constraints, the PCR products were not sent for sequencing to obtain their accession numbers.
